# Effects of APOE Alleles and Productive Aging Activities on Cognitive Health Among Older Hispanics

**DOI:** 10.1093/geroni/igaf122.1076

**Published:** 2025-12-31

**Authors:** Ernest Gonzales, Jane Lee, Cliff Whetung

**Affiliations:** New York University, New York, New York, United States; University of Hawaiʻi at Mānoa, Honolulu, Hawaii, United States; University of Minnesota Duluth, Duluth, Minnesota, United States

## Abstract

This study examined the longitudinal associations of productive aging activities – formal education, employment, volunteering – with cognitive functioning in the context of genetic inheritance (APOE alleles) among older Hispanics in the United States. Mixed effect growth curve models tested associations with respondents in the Health and Retirement Study (8,558 observations longitudinally from 2010 to 2020), controlling for known health, economic, and social covariates. A fifth of respondents carried one or two ε4 alleles. Nearly half of respondents had less than a high school diploma (46%), nearly half worked for pay (45%), approximately one quarter engaged in formal volunteering (24%), and approximately a third engaged in informal volunteering (36%). Respondents with ε4 alleles experienced lower levels of cognitive health at baseline, yet slower slopes of cognitive decline across models. Higher levels of education conferred greater cognitive health cross-sectionally and a slower cognitive decline rate over time. Although employed respondents reported lower levels of cognitive health at baseline, employment protected their cognitive health overtime relative to Hispanics not in the labor force. Formal volunteering resulted in higher levels of cognitive health, yet associations were unstable across models. Foreign born Hispanics experienced higher levels of cognitive health at baseline relative to US born Hispanics. Income, depression, and other social contexts influenced cognitive health at baseline and overtime in expected directions. Productive activities contributed to cognitive health, above and beyond genetic factors. Productive aging, income, and health policies and programs can bolster cognitive health. We will discuss areas for further research.

